# Seed‐specific RNAi in safflower generates a superhigh oleic oil with extended oxidative stability

**DOI:** 10.1111/pbi.12915

**Published:** 2018-04-02

**Authors:** Craig C. Wood, Shoko Okada, Matthew C. Taylor, Amratha Menon, Anu Mathew, Darren Cullerne, Stuart J. Stephen, Robert S. Allen, Xue‐Rong Zhou, Qing Liu, John G. Oakeshott, Surinder P. Singh, Allan G. Green

**Affiliations:** ^1^ CSIRO Agriculture and Food Canberra ACT Australia; ^2^ CSIRO Land and Water Acton ACT Australia

**Keywords:** safflower, GM crop, oleochemical, small RNA, field trials, oxidative stability

## Abstract

Vegetable oils extracted from oilseeds are an important component of foods, but are also used in a range of high value oleochemical applications. Despite being biodegradable, nontoxic and renewable current plant oils suffer from the presence of residual polyunsaturated fatty acids that are prone to free radical formation that limit their oxidative stability, and consequently shelf life and functionality. Many decades of plant breeding have been successful in raising the oleic content to ~90%, but have come at the expense of overall field performance, including poor yields. Here, we engineer superhigh oleic (SHO) safflower producing a seed oil with 93% oleic generated from seed produced in multisite field trials spanning five generations. SHO safflower oil is the result of seed‐specific hairpin‐based RNA interference of two safflower lipid biosynthetic genes, *FAD2.2* and *FATB*, producing seed oil containing less than 1.5% polyunsaturates and only 4% saturates but with no impact on lipid profiles of leaves and roots. Transgenic SHO events were compared to non‐GM safflower in multisite trial plots with a wide range of growing season conditions, which showed no evidence of impact on seed yield. The oxidative stability of the field‐grown SHO oil produced from various sites was 50 h at 110°C compared to 13 h for conventional ~80% oleic safflower oils. SHO safflower produces a uniquely stable vegetable oil across different field conditions that can provide the scale of production that is required for meeting the global demands for high stability oils in food and the oleochemical industry.

## Introduction

Plant oils are primarily used in foods, but it is less well known that vegetable oils are also the largest renewable feedstocks for chemical industries (Biermann *et al*., 2011). These oleochemical industries are growing to meet the global demand for sustainable, environmentally sensitive products, including surfactants, emollients, polymers and lubricants (Alam *et al*., 2014; Salimon *et al*., 2012). In the lubricant sector, oleochemical replacements are sought to replace the nonrenewable petrochemicals that, when lost to the environment, cause pollution requiring remediation of soil and water (Fofana, 2013; Garces *et al*., 2011). A significant drawback to the wider adoption of vegetable oils as lubricants is their poor oxidative stability that limits shelf‐life and functionality (Fofana, 2013). The physical and chemical characteristics of oils, such as oxidative stability and freezing point, are largely determined by their fatty acid profile (Gunstone, 2003; Kockritz and Martin, 2008). Generally, oils high in saturated fatty acids are resistant to oxidation, but are solid at ambient temperatures, whereas oils high in polyunsaturates remain liquid at cool temperatures, but suffer from oxidative damage and free radical attack. It has long been recognized that oils rich in monounsaturated oleic acid offer a unique combination of good oxidative stability and lubricity at cool temperatures (Kinney, 1994; Vanhercke *et al*., 2013). In this report, we focus on the production of a plant oil containing near‐pure levels of oleic acid, while reducing polyunsaturated and saturated fatty acids to extremely low levels.

Since the late 1950's plant biotechnologists have altered the composition of oleic acid in oilseeds from a base of 10%–30% oleic to the 70%–90% range, including canola (Stoutjesdijk *et al*., 2000), soya bean (Buhr *et al*., 2002; Haun *et al*., 2014), cotton (Green *et al*., 2002), peanut (Jung *et al*., 2000), flax (Chen *et al*., 2015), crambe (Li *et al*., 2016), camelina (Nguyen *et al*., 2013), sunflower (Alberio *et al*., 2016; Lacombe *et al*., 2009) and safflower (Horowitz and Winter, 1957; Weisker, 1997). Some approaches have relied upon genomic level modification via mutagenesis or targeted genome editing (Haun *et al*., 2014) which potentially impacts fatty acid metabolism in all parts of the plant and, where tested, also results in undesirable pleiotropic impacts on crop performance (Alberio *et al*., 2016; Weisker, 1997). More targeted transgenic approaches using seed‐specific silencing constructs have resulted in very high oleic acid levels in seed oils but fail to completely remove polyunsaturated linolenic acid, C18:3 (Buhr *et al*., 2002; Li *et al*., 2016; Nguyen *et al*., 2013; Stoutjesdijk *et al*., 2000), that contributes to poor oxidative stability. In these respects, a broadacre oilseed crop capable of producing oil with over 90% oleic, with no linolenic acid, has yet to be developed.

## Results

**Figure 1:**
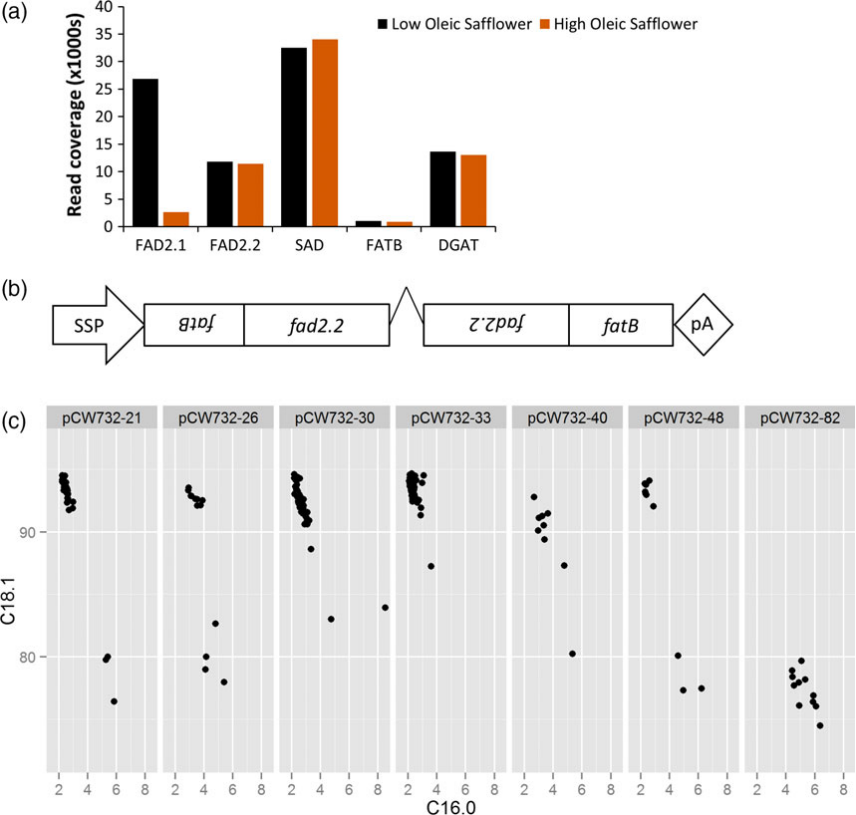
Generation of SHO events in safflower. (a) expression profile of key genes in developing embryos of safflower varieties, low oleic safflower (LO) with 80% linoleic acid, and high oleic safflower (HO) with 80% oleic acid. Stearic acid desaturase (SAD) and diacylglycerolacyltransferase (DGAT) are included as representative genes that are not altered in expression levels; (b) schematic of the transgenic construct, pCW732, indicating the seed‐specific promoter (SSP, linin) and the orientation and length of the two gene fragments used to generate a hairpin RNA targeting *CtFAD2.2* and *CtFATB*. The length of the fragments used for the hairpin RNA is 780 and 414 bp for *CtFAD2.2* and the *CtFATB*, respectively; (c) a representative sample of transgenic events at the T2 stage, pCW732‐21, pCW732‐26, pCW732‐30, pCW732‐33, pCW732‐40 and pCW732‐48 using the percentage of oleic acid (C18.1) and palmitic acid (C16.0). These events were selected from other 30 initial events that show a similar ‘SHO profile’. The panel includes ‘pCW732‐82’, an event that escaped selection as a false positive but is maintained as a null (~80% C18.1, ~5% C16:0) for illustrative purposes. Each individual fatty acid profile from the T2 seed is displayed as a single point, and different T0 plants produced different numbers of T2 seed.

We chose safflower (*Carthamus tinctorius L*.) as the crop platform for the development of superhigh oleic oils. Safflower has the particular advantage over canola or soya bean of containing no C18:3 in the seed oil, and this feature simplifies the genetic interventions required to improve the overall oxidative stability. The genetic and biochemical basis of lipid and oil biogenesis in safflower seed is well understood (Cao *et al*., 2012; Guan *et al*., 2012; Jones *et al*., 1995; Knutzon *et al*., 1992; Stymne and Appelqvist, 1978). The flux of lipid synthesis towards oleic acid can be prematurely diverted via the activity of FATB, a thioesterase transporting 16:0 from the chloroplast to the cytoplasm where it accumulates into oil. Oleic acid produced in the chloroplast is exported to the cytoplasm via another class of thioesterases, FATA, and is made available for desaturation to linoleic acid, C18:2, via fatty acid desaturases (FAD2 enzymes) operating on phosphatidylcholine (PC) in the endoplasmic reticulum. Current safflower varieties contain seed oils with two major classes of oil profile, either low oleic (LO; ~13% oleic acid/78% linoleic) or high oleic (HO; ~78% oleic acid/13% linoleic). This major change in the ratio of oleic to linoleic is due to a mutation in *CtFAD2.1* (Liu *et al*., 2013), an oleoyl desaturase functioning as a major contributor to the production of linoleic acid in the seed oil. We have previously found that developing seeds of HO safflower express one further oleoyl desaturase, CtFAD2.2 (Cao *et al*., 2012) and that this gene is therefore the likely candidate for producing the remaining linoleic acid in HO safflower. We also expected that further increases in the flux towards oleic acid could be achieved by simultaneously reducing the activity of FATB, a strategy used previously in soya bean (Buhr *et al*., 2002). We investigated the expression of these genes by a deep sequence analysis of RNA extracted from developing embryos of LO and HO varieties. This analysis confirmed that *CtFAD2.1* transcript was reduced in HO safflower relative to LO safflower; however, *CtFAD2.2* transcript abundance remained unchanged (Figure 1a). This approach also found a single transcript with homology to the family of saturated palmitic acid thioesterases, here named *CtFATB*, and the abundance of this transcript was unchanged between HO and LO safflower. These results suggest that knock‐down of both CtFAD2.2 and CtFATB activities in a HO safflower background could reduce production of linoleic and palmitic acids and increase the flux of lipids into oleic acid during seed development.

**Figure 2:**
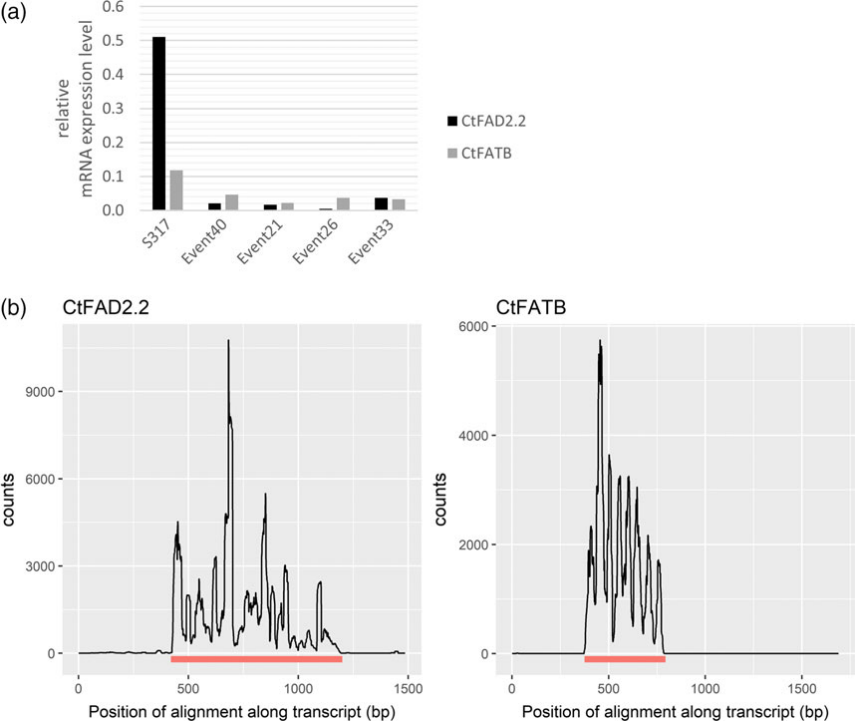
Expression of *CtFAD2.2* and *CtFATB* in developing seeds of safflower. (a) qPCR analysis of *CtFAD2.2* and *CtFATB*; (b) alignment of small RNA (21–24 nt long) against templates for *CtFAD2.2* and *CtFATB*. A wild‐type HO line produced no sRNA against these templates. The bar underneath each graphic for either gene indicates the exact length and location of the template used for the hairpin RNAi construct in pCW732 relative to the transcript.

While there are a range of strategies available to silence genes, we chose an approach that was designed to reduce the activity of two genes in the developing seed yet avoiding their knock‐down in other nonseed tissues, similar to our previous experience in Arabidopsis (Smith *et al*., 2000), flax (Chen *et al*., 2015) and cotton (Green *et al*., 2000). A TDNA‐based plant transformation construct was designed which included a seed‐specific linin promoter (Nykiforuk *et al*., 2012) driving the expression of an intron‐mediated hairpin silencing molecule that included a 414‐bp fragment of *CtFATB* and another 780‐bp fragment of *CtFAD2.2*, here termed pCW732 (Figure 1b). This construct was transformed into a HO oleic variety of safflower and over 30 independent transgenic events were created. The first generation of seeds formed on the primary transgenic plants (here termed T2 seed) was nondestructively analysed for oil profile (Figure 1c). This analysis found that the lipid composition profiles fell into two classes, either the 75%–82% range of oleic characteristic of the non‐GM parent or a new range with a composition of 90%–94% oleic acid, 1%–1.5% linoleic acid with palmitic acid acids reduced from ~5% to 2%. We termed the latter class as superhigh oleic (SHO) safflower. Real‐time PCR analysis on four events (pCW732‐21, 26, 33 and 40) found that the expression of *CtFAD2.2* and *CtFATB* in developing embryos of SHO lines was markedly reduced relative to wild type (Figure 2a). Deep sequencing analysis confirmed the presence of small RNA generated by pCW732 that map against both *CtFAD2.2* and *CtFATB* precisely within the confines of the hairpin DNA sequence of pCW732 (Figure 2b). Events 21, 26, 33 and 40 were then backcrossed into HO safflower and the resulting F2 seed displayed a ratio of SHO:HO consistent with a single and dominant TDNA locus for Events 21, 26 and 40 and two independent loci for Event 33 (data not shown).

**Figure 3:**
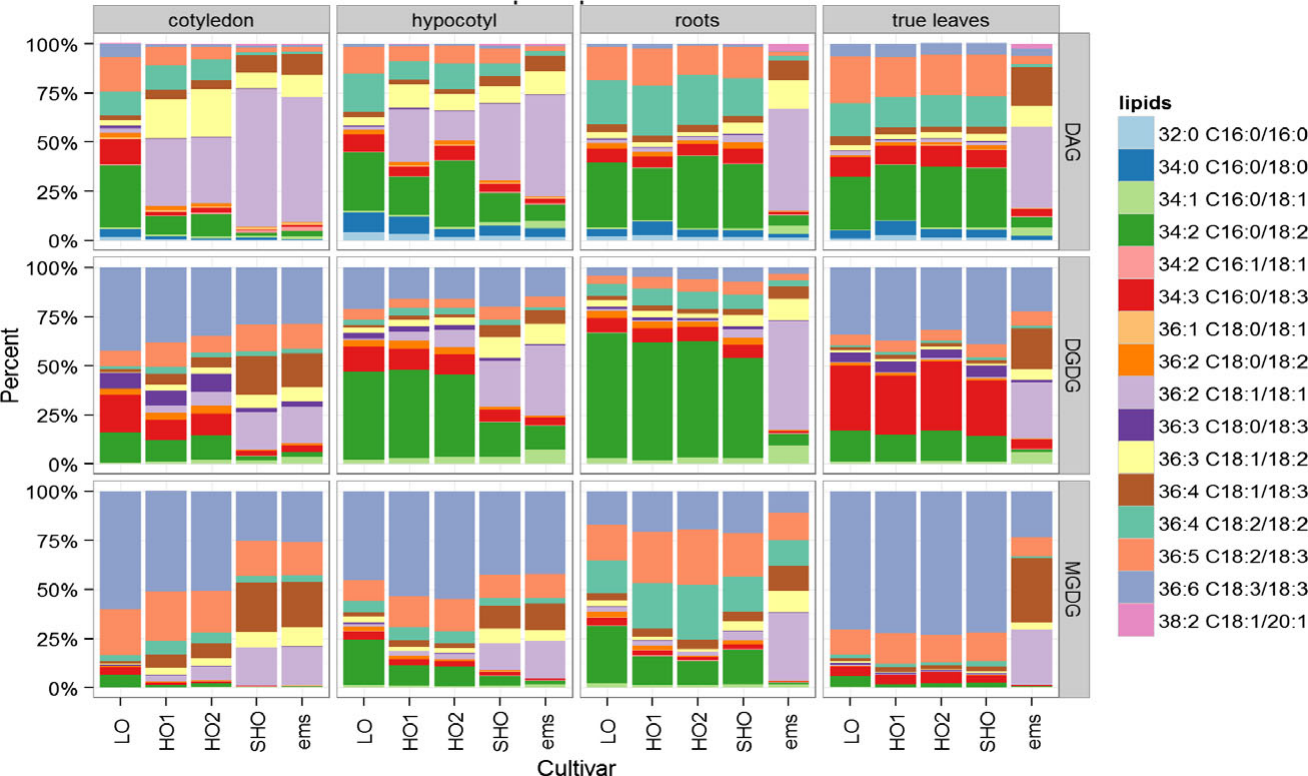
Profiling membrane‐associated lipid species of different developmental stages from SHO and non‐GM safflower varieties. The analysis includes varieties that have altered seed oleic profiles, such as low oleic (LO; Centennial), high oleic (HO1, S317; HO2, null segregant), superhigh oleic (SHO, Event 26) and S901 (ems). Other SHO lines were analysed and display similar trends to Event 26. Diacylglycerol (DAG), digalactosyldiacylglycerol (DGDG) and monogalactosyldiacylglycerol (MGDG).

A comprehensive lipidomic analysis of seed and nonseed tissues in SHO lines and in other available safflower varieties was conducted. Alongside the LO and HO safflower, we also included a HO‐derived ethyl methanesulphonate (ems/S901) mutant of safflower, with very high oleic acid content in the seed (~90%), but compromised yield (Weisker, 1997). This analysis found that in roots and true leaves, the dominant lipid species such as diacylglycerol (DAG), digalactosyldiacylglyercol (DGDG) and monogalactosyldiacylglyercol (MGDG) were unchanged between LO, HO and SHO lines (Figure 3), and showed the high polyunsaturated fatty acid composition typical of these vegetative tissues. In contrast, ems/S901 contained a marked increase in the monounsaturated content relative to the polysaturated fatty acids in all tissues sampled. These results indicate that the transgenic RNA interference approach encoded by pCW732 used in generating SHO is restricted to seed and developmentally derived organs, such as the emergent cotyledons and hypocotyls.

Field trials of transgenic safflower events were conducted to determine whether SHO levels in seed oil can be achieved outside of a laboratory setting and also to generate sufficient materials for functional testing of the resulting oil. From a handful of elite SHO events (Figure 1c), Event 33 was chosen for the production of larger quantities of SHO seed due to the presence of two active transgene alleles and reduced incidence of HO safflower segregants occurring at early stages of event selection. Non‐GM safflower varieties and Event 33 seed were grown in field trials located in Kununurra, Western Australia (fourth‐generation seed), and Narrabri, northern New South Wales (fifth‐generation seed; see Figure S1). Field‐grown seed were analysed, both as single seed and as bulk samples. Single seed analyses of Event 33 confirmed that the lipid profiles were similar to that found in laboratory settings (~93% oleic, 1.5% linoleic, 2% palmitic). Bulk samples of field‐grown seed (150 g or ~3000 seed per sample) were crushed to produce oil samples for functional testing, including oxidative stability (Table 1). This analysis found that oils extracted from Event 33 grown in two different field trials displayed a fourfold improvement in the oxidative stability index (OSI) of 50 h (performed at 110°C) relative to the oil of HO, at 13 h. Our results indicate that SHO is at least twice as stable as a range of high and very high oleic seed oils previously assessed under identical conditions (Merrill *et al*., 2008), with the highest value reported previously being 18.5 h for very high oleic canola oil. Safflower seed also produce a range of tocopherols that were unchanged in the SHO lines compared to non‐GM parent varieties, and therefore, these antioxidants are not contributing to the improved stability of SHO relative to high oleic safflower oil.

**Table 1:** Fatty acid profile and oxidative stability of safflower oils extracted from field‐grown safflower

Analysis	Common name	Event‐location			
		LO‐KRS	HO‐KRS	E33‐KRS	E33‐ACRI
Fatty acid (per cent of total)					
C14	Myristic	0.1	0.1	0.0	0.0
C16	Palmitic	6.9	4.9	2.5	2.3
C16:1n7	Palmitoleic	0.1	0.1	0.2	0.2
C18	Stearic	2.6	0.6	0.6	0.1
C18:1n9	Oleic	12.8	75.4	92.1	93.2
C18:1	Octadecenoic	0.6	0.2	0.1	0.1
C18:2	Linoleic	75.3	15.1	1.1	1.2
C18:3n3	Alpha‐Linolenic	0.1	0.1	0.1	0.1
C20	Arachidic	0.4	0.5	0.5	0.3
C20:1	Eicosenoic	0.2	0.3	0.4	0.4
C20:3n3	Eicosatrienoic	0.1	0.0	0.0	0.0
C20:4n3	Eicosatetraenoic	0.1	0.0	0.0	0.0
C22	Behenic	0.2	0.3	0.3	0.3
C24	Lignoceric	0.1	0.2	0.2	0.2
C24:1n9	Nervonic	0.1	0.2	0.2	0.1
Others		0.3	2.1	1.9	1.4
Total saturates		10.3	6.5	4.1	3.3
Total monounsaturates		13.9	76.2	92.8	94.0
Total polyunsaturates		75.5	15.2	1.2	1.3
Total omega3		0.2	0.1	0.1	0.1
Total omega 6		75.3	15.1	1.1	1.2
Total omega 9		13.2	75.9	92.6	93.7
Tocopherols and sterols (mg/100 g)					
DeltaTocopherol		4	5	4	3
GammaTocopherol		2	1	2	2
AlphaTocopherol		58	58	58	58
Campesterol		39	51	60	66
Stigmasterol		19	21	20	21
B‐sitosterol		132	184	185	188
Other		234	265	207	174
Stability and viscosity					
OSI (Rancimat@110°C in hours)		3.2	12.8	50.8	48.2
Viscosity (kinematic@40°C in cSt)		29.2	36.8	39.1	39.2

Analysis of safflower seed oils isolated from ~3000 seed batches from two different field sites (Kununurra, KRS; Narrabri, ACRI). The SHO event is pCW732‐33, E33, and is the fourth generation at Kununurra‐KRS and the fifth generation at Narrabri‐ACRI. Conventional safflower varieties are high oleic (HO) variety and low oleic (LO) varieties. All data are presented as an average of two independent technical repeats by independent commercial analysis and are representative of more extensive testing.

**Figure 4:**
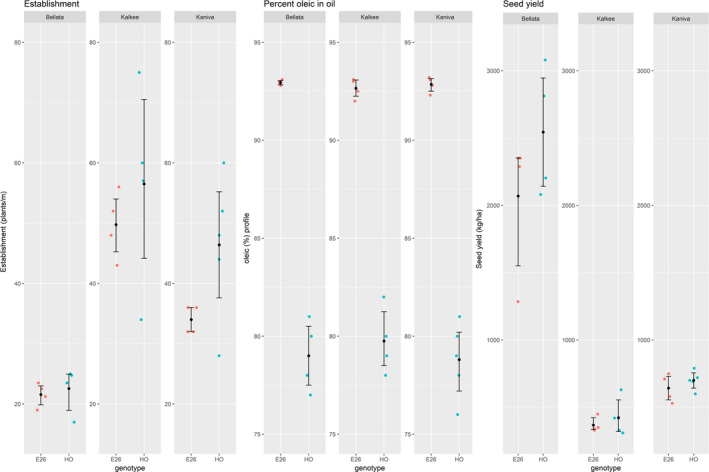
Performance of an elite SHO event and wild type in three field trials. Field trials were conducted at Bellata, northern New South Wales, and Kalkee and Kaniva, close to Horsham in western Victoria (see Figure S2 for details). SHO Event 26, E26; HO is a non‐GM parent. All data presented including individual data as single points and the summary statistics of the mean and 95% confidence intervals calculated using nonparametric bootstrapping methods within the Hmisc package in R.

Three field trials were conducted in the 2015–2016 growing season to assess the yield and other key parameters of an elite SHO event, Event 26, relative to its null segregant HO. For these trials, Event 26 seed was sourced from the Narrabri trial and was therefore the sixth generation (See Figure S1), and was chosen due to a combination of single locus TDNA insertion and the availability of seed. Trial sites were chosen in the districts of Bellata, Kalkee and Kaniva spanning significant cereal cropping regions of the eastern cropping belt of Australia (Figure S2) and all trials included four replicates of small plots of E26 sown alongside conventional safflower varieties. All field trials were rainfed with those based in Bellata, northern New South Wales, receiving a favourable 300 mm of in‐season rainfall, and the sites in western Victoria, Kalkee and Kaniva, receiving only 100 mm of in‐season rainfall. The establishment of plants in field conditions was statistically different between the HO and Event 26 lines at the Kaniva site but was not statistically different at Kalkee or Bellata (Figure 4), indicating that the dramatic change in the seed oil profile of SHO compared to HO may impact the vigour of the seedlings under some field conditions. Despite differences in establishment, the overall seed yields at the end of the growing season for Event 26 were not significantly different to HO at the three sites, with both GM and non‐GM lines yielding over 2000 kg/ha at Bellata, whereas the much drier sites at Kalkee and Kaniva yielded approximately 500 kg/ha. Despite these different locations and rainfall conditions, these three trials all produced seed oil with SHO profiles (~93% oleic) for Event 26, well above the HO parent at 75%–80%, and consistent with the previous four generations. These field trials demonstrate that the seed‐specific silencing of two genes in safflower produces superhigh oleic oil under contrasting environmental conditions without negatively impacting the overall yield of the crop. More thorough multisite and multiyear testing is being conducted with a range of elite SHO events to more accurately assess the impact of the introduced transgene on the agronomy and performance of SHO relative to conventional varieties.

## Discussion

In this report, we detail a renewable source of oils comprised almost exclusively of oleic acid. This result builds upon a large number of studies spanning decades of plant breeding aimed at elevating oleic content in seed at the expense of polyunsaturates and saturated fatty acids. From both laboratory and field trial seeds, the SHO oil profile can be summarized as approximately 93% monounsaturates, 1.5% polyunsaturates and 4% saturates. The SHO trait has proven stable over five generations of growth, with three of these cycles in sequential field trials. To our knowledge, this is the first such demonstration of the sustained performance of hairpin‐based silencing under these multiseason conditions. The hairpin‐based silencing approach used here is different from other silencing techniques. For instance, the seed‐specific silencing of FAD2 and FATB in soya bean uses a seed‐specific promoter to express a ribozyme termination technology and has been demonstrated to be stable over numerous generations, including for commercial release (Buhr *et al*., 2002; Graef *et al*., 2009). Inactivation of gene activity via direct gene editing will also result in a stably inherited trait (Haun *et al*., 2014); however, direct editing of the FAD2 and FATB genes can also alter the lipid profiles in nonseed tissues, a change that can have implications for overall crop performance.

Although safflower is currently a niche crop in Australia, it is grown commercially in over 60 countries (Chapman *et al*., 2010; Nykiforuk *et al*., 2012) and has varieties well suited to a range of growing conditions from subtropical to cool temperate. We calculate that modest acreages of SHO safflower could generate sufficient high stability oil to begin the replacement of petrochemical‐based lubricants with a vegetable‐based feedstock. Recently highly stable, oleic‐rich oils have been developed in algal fermentation systems (Franklin *et al*., 2013; Szabo *et al*., 2014) containing ~89% oleic acid and 0.5% polyunsaturates and an oxidative stability ~56 h at 110°C. Crop and fermentation platforms should be seen as two complementary approaches to meet the global demands for high stability oils, with alternative supplies needed to cover gaps in production. Nevertheless, the SHO safflower described here is poised to produce the lowest cost and purest source of oleic‐rich oil at scales that could be compatible with some existing petrochemical product volumes.

Oleic acid is an excellent starting point for other engineered oils with applications in the chemical industry. Firstly, oleic acid is a substrate for metabolic pathways that produce a range of other industrially useful products, such as branched‐chain fatty acids (Yu *et al*., 2014), ricinoleic acid (Vandeloo *et al*., 1995) and waxes (Lardizabal *et al*., 2000). All of these industrial fatty acids would benefit from elevated levels of oleic in SHO but also the lack of unstable polyunsaturates that can influence the overall utility of the extracted products. Therefore, SHO safflower offers the opportunity to produce other oleochemicals *in planta* to produce novel engineered feedstocks. Secondly, there has been an emergence of metathesis reagents (Marx *et al*., 2015) and allied green chemistries (Biermann and Metzger, 2008, 2014) that can convert oleic acid into a range of derived products. In these reactions, large volumes of near‐pure oleic acid could facilitate the development of affordable and renewable products.

## Experimental procedures

### Plant materials and genetic transformation

Safflower varieties S317 (PI 599253) and Centennial (PI 538779) were obtained from the USDA ARS collection. S901 was obtained from the ATCC collection (ATCC 209181). The high oleic safflower used for genetic transformation is an advanced breeding line being developed for commercial release and was transformed with pCW732 using an Agrobacterium‐mediated method as previously described (Belide *et al*., 2011). Manual crossing of safflower plants was performed by standard methods (Mayerhofer *et al*., 2011).

### Deep sequencing for transcriptomic and small RNA analysis

Total RNA was extracted from developing seeds of S317 (high oleic, HO variety) and Centennial (low oleic, LO variety) at 10, 15 and 20 days after flowering (DAF), a period during which most oil synthesis occurs in safflower, and prepared for sequencing using Illumina TruSeq Sample Prep Kit according to manufacturer's instructions. Libraries were individually barcoded and sequenced on an Illumina HiSeq2000 platform generating 100‐bp paired end reads. After quality control of reads, transcriptomic data of either S317 (DAF 10, 15, 20) and Centennial (DAF 10, 15, 20) were pooled and assembled into *de novo* assemblies of transcriptomes from either variety. These assemblies confirmed the sequences of the large *FAD2* family in safflower (Cao *et al*., 2013), the mutation in *FAD2.1* in S317 (Liu *et al*., 2013), and generated a putative palmitic acid thioesterase sequence *CtFATB* (NCBI Accession number KU059745). Differential expression analysis was conducted using alignment of reads on template assemblies using a pairwise comparison of HO and LO at each time point of oil accumulation using BioKanga software (http//sourceforge.net/projects/biokanga/files/).

For small RNA analysis, total RNA was extracted from maturing safflower seed using PureLink Plant RNA Reagent (Thermo Fisher Scientific) with the following modifications to the manufacturer's protocol; (i) the chloroform extraction was repeated. (ii) Precipitation of RNA was carried out overnight at −20°C. RQ1 DNAse (Promega; http://promega.com/) was used to treat RNA. The RNA was subjected to commercial deep sequencing using the Illumina TruSeq small RNA Sample Prep Kit and Illumina‐based 100‐bp single read technologies (John Curtin School of Medical Research, Canberra). sRNAs were trimmed of adaptor sequences using Trimmimatic (Bolger *et al*., 2014) and were back‐aligned to template sequences of *CtFAD2.2* and *CtFATB* using ShortStack (Axtell, 2013), allowing a single mismatch. Locations with a minimum unique read coverage of five are reported**.**

### Transcriptomic analysis via real‐time PCR

Total RNA was extracted from maturing safflower seed as outlined above; however, the precipitated RNA was further cleaned using Plant RNAeasy columns (Qiagen) of small RNA. cDNA synthesis was carried out using Superscript III reverse transcriptase (Thermo Fisher Scientific) according to the manufacturer's protocol with an oligo dT primer (Thermo Fisher Scientific). For each RNA sample, three separate cDNA synthesis reactions were carried out. Real‐time quantitative (qRT)‐PCR was carried out as described (Allen *et al*., 2007). Primers used in this analysis are outlined in Table S1.

### Construction of pCW732

A 414‐bp fragment of *CtFATB* was amplified from a cDNA library isolated from S317 with Not1 flanking sites and ligated into the Not1 site of pENTR‐Topo (Thermo Fisher Scientific), producing pCW700. Similarly, a 780‐bp product of *CtFAD2.2* was generated to include flanking AscI sites and cloned into the AscI site of pCW700, producing pCW701. The orientation of the *CtFATB* and *CtFAD2.2* fragments was designed to be tail to tail, as outlined in Figure 1b. Gateway cloning techniques were used to introduce the *CtFATB‐CtFAD2.2* fragment into a hairpin‐based vector where the plant selectable marker was hygromycin resistance and the promoter driving expression of the RNAi hairpin was the linin promoter (Chaudhary *et al*., 2004), generating pCW732.

### Precise mapping of genome location of TDNA events

The flanking gDNA sequences next to TDNA insertion sites were investigated using the Universal GenomeWalker™ 2.0 kit (Clontech, Mountain View, CA) according to manufacturer's instructions. In brief, isolated gDNA was digested overnight with DraI and EcoRV, and subsequently ligated with an adaptor. This was used as a template for primary and nested PCR using adaptor primers and gene TDNA‐specific primers (data not shown). PCR products were sequenced to determine the TDNA and flanking gDNA sequence using a draft safflower genomic database. From the flanking sequence information, event‐specific primers (data not shown) were designed to develop a PCR‐based screening method to determine the zygosity of the inserted TDNA for each event. Zygosity testing was used throughout field trials for selection of homozygous plants.

### Field trials of SHO

Field trials spanned 3 years (2014–2015–2016) and five different sites across Australia (See Figure S1 and S2). In 2014, a selection of SHO events, pCW732‐21, pCW732‐26, pCW732‐30, pCW732‐33, pCW732‐40 and pCW732‐48, were grown under irrigated conditions at Kununurra, in northern Western Australia (OGTR website, DIR121 (http://www.ogtr.gov.au/internet/ogtr/publishing.nsf/Content/DIR121, accessed September 2015)) conducted between May 2014 and September 2014. A subset of these materials (Events pCW732‐21, pCW732‐26, pCW732‐30, pCW732‐33 and pCW732‐40) were grown at second site in Narrabri, New South Wales (OGTR website, DIR121 (http://www.ogtr.gov.au/internet/ogtr/publishing.nsf/Content/DIR121, accessed September 2015)). In 2015, three small plot trials were established to assess yield and other performance parameters; Bellata, northern New South Wales, and two sites, Kalkee and Kaniva, both within 50 kilometres of Horsham, western Victoria (OGTR website, DIR131 (http://www.ogtr.gov.au/internet/ogtr/publishing.nsf/Content/DIR131, accessed September 2015)). Seeds were direct drilled into prepared seed beds in 17 × 2 m small plots with four replicates per treatment per trial following randomized designs. In all trials, single plant and bulk plot harvests were undertaken, either for pure seed production or bulk up for oil crushing. At all sites, a range of wild‐type safflower varieties were grown as comparator plots, including S317, Centennial and S901, and null segregants that were generated from tissue culture. SHO events and wild‐type safflower plots were arranged in four replicates using standard randomized designs with a buffer row of non‐GM safflower. All seeds for yield assessments were harvested using small plot harvesters. Zygosity testing was used throughout field trials for selection of homozygous plants.

### Statistical treatments

Statistics were generated using the Hmisc package (https://cran.r-project.org/web/packages/Hmisc/Hmisc.pdf) within software package ggplot within R (http://ggplot2.tidyverse.org/reference/index.html). Statistical comparisons were generated by calculating the 95% confidence intervals of a population of data using nonparametric bootstrapping methods with 100 iterative calculations. Different treatments with overlapping confidence intervals are considered to be not significantly different, where required individual data points were plotted using slight jitter to avoid overplotting.

### Preparation of oil from field‐grown seed and analysis

Field‐grown materials were dried at 37°C for a week and crude oils extracted using a benchtop cold oil presser. The extracted crude oil was centrifuged at 10 000 ***g*** for 10 min, and the supernatant was filtered through a 40‐μm membrane and flushed with nitrogen gas prior to storage and analysis. 50 mL samples were used for independent commercial analysis (POS‐BioSciences, Saskatchewan, Canada). The fatty acid profile was determined using AOAC 969.33 and AOAC 996.06, and the oxidative stability index (OSI at 110°C) was conducted according to AOCS Cd 12b‐92 and the tocopherol and sterol profiles as previously described (Slover *et al*., 1983).

### Nondestructive analysis of lipid profile of safflower seed by gas chromatography

Safflower seeds were imbibed on wet filter paper overnight (16 h) at room temperature to allow the seed coat to be removed. The tip of cotyledons (5 mm) was sampled for fatty acid methyl ester (FAME) analysis and the remaining seed grown to maturity. FAMEs were prepared essentially as previously described (Zhou *et al*., 2013), with slight modifications. The methylation was extended to 4 h with 800 μL 1N methanolic‐HCl (Supelco, Bellefonte,PA). Gas chromatography analysis with flame ionization detector was performed essentially as previously described (Zhou *et al*., 2013), except the ramping programme was changed to an initial temperature at 150°C holding for 1 min, then raised to 180°C at 10°C/min and to 240°C at 50°C/min holding for 4 min. GLC standard 411 (Nuchek Prep Inc., Elysain, MN) was used for calibration.

### Total lipid extraction for lipidomic analysis

Total lipids were extracted from freeze‐dried cotyledon, hypocotyl, roots and true leaves of two‐week‐old safflower varieties. Freeze‐dried leaf tissue was ground to powder in a microcentrifuge tube containing a metallic ball using Reicht tissue lyser (Qiagen) for 3 min at 20 frequency/s. Chloroform:methanol (2:1, v/v) was added and mixed for a further 3 min before the addition of 1:3 (v/v) of 0.1 m KCl. The sample was then mixed for a further 3 min before centrifugation (5 min at 14 000 ***g***), after which the lower lipid phase was collected. The remaining phase was washed once with chloroform, and the lower phase extracted and pooled with the earlier extract. Lipid phase solvent was then evaporated completely using N_2_ gas flow and the lipids resuspended to 20 mg/mL chloroform per mg of the extracted oil.

### Lipidomic analysis via liquid chromatography‐mass spectrometry

Lipids extracts were diluted in 1:100 mL butanol:methanol (1:1, v/v) and analysed by liquid chromatography‐mass spectrometry (LC‐MS), based on previously described methods (Reynolds *et al*., 2015). Briefly, an Agilent 1290 series LC and 6490 triple quadrupole MS with Jet Stream ionization was used for all analyses. The phosphatidylcholine (PC) and lysophosphatidylcholine (LPC) species were separated on an Agilent 120 HILIC column (2.1 × 100 mm, 2.7 μm), over a gradient from 95% acetonitrile to 75% acetonitrile with 20 mm ammonium acetate. PC and LPC hydrogen adducts were quantified by the characteristic 184 *m/z* phosphatidyl head group ion under positive ionization mode. The ammonium adducts of monogalactosyl diacylglycerol (MGDG), digalactosyl diacylglycerol (DGDG), diacylglycerol (DAG) and TAG lipid species were analysed by the neutral loss of singular fatty acids C_16_ to C_20_. Multiple reaction monitoring (MRM) lists were based on the following major fatty acids: 16:0, 16:3, 18:0, 18:1, 18:2, 18:3, using a collision energy of 28 V, and chromatographically separated using an Agilent Poroshell column (50 mm × 2.1 mm, 2.7 μm) and a binary gradient with a flow rate of 0.2 mL/min. The mobile phases were as follows: A. 10 mm ammonium formate in H_2_O:acetonitrile: isopropanol (5:45:50, v/v); B. 10 mm ammonium formate in H_2_O:acetonitril: isopropanol (5:20:75, v/v). Individual MRM TAG was identified based on ammoniated precursor ion and product ion from neutral loss. Results were integrated using Agilent Mass Hunter Quantitative software and exported into R for statistical and graphical analysis.

### High‐throughput lipidomic analysis of safflower seeds

Individual seeds were crushed between layers of filter paper discs. The oil was extracted from the discs using 200 μL of a 1:1 solution of methanol:butanol with butylated hydroxytoluene (25 mg/L). Samples were further diluted 1:200 into mobile phase, 80:15:5 isopropanol:acetonitrile:water with 10 mm ammonium formate, and separated by an isocratic elution on an Agilent Zorbax XDB C18, 1.8 μm 2.1 × 30 mm, cartridge at 0.3 mL/min. TAG species monitored were identified by analysis of representative oil samples of each safflower line using an Agilent 6550 jet stream Q‐TOF. Quantitative lipid analysis of seed samples was conducted on an Agilent 6490 triple quadrupole mass spectrometer. Multiple reaction monitoring (MRM) of the neutral loss of acyl chains (from C14 to C24 with 0–3 sites of desaturation) was conducted upon TAG lipid species ranging from 50 to 60 carbons. A total of 54 MRMs were analysed to obtain a complete oil profile. Results were first analysed in Mass Hunter Quantitative Analysis (Agilent) and then exported to further analysis using R studio.

## Supporting information


**Figure S1** A schematic outlining the biogenesis of SHO Events outlined in this report, using Event 26 as an example.
**Figure S2** Location of field trials for the production of superhigh oleic safflower.
**Table S1** Primers used for RT Q‐PCR.
