# Supplementary material for: Seed‐specific RNAi in safflower generates a superhigh oleic oil with extended oxidative stability
Source: Plant Biotechnol J. 2018 Apr 2;16(10):1788–96. doi: 10.1111/pbi.12915 (PMC6131418; doi:10.1111/pbi.12915)
Supplement: Supplementary file 1 — Figure S1 A schematic outlining the biogenesis of SHO Events outlined in this report, using Event 26 as an example. Figure S2 Location of field trials for the production of superhigh oleic safflower. Table S1 Primers used for RT Q‐PCR. [file PBI-16-1788-s001.docx]

| Supplementary Table 1: Primers used for RT Q-PCR | | | |
| --- | --- | --- | --- |
|  |  |  |  |
| Primer Name | Sequence 5’ 🡪 3’ | Tm value | Base |
| qctFAD6-C-A2 | GTTCCAACAATATCTTCCACCAGT | 63.8 | 24 |
| qctFAD6-C-S2 | CATTGAAGTCGGTATTGATATCTG | 61.1 | 24 |
| qctROD-A1 | CGATGGTGTAGTGTCCTCTTGA | 64.0 | 22 |
| qctROD-S1 | GTATTCAACTCAGCTTCCATTGC | 63.6 | 23 |
| qctFatB-C-a1 | TTCTTGGGACATGTGACGTAGAA | 65.4 | 23 |
| qctFatB-C-s1 | CCTCACTCTGGGACCAAGAAAT | 65.3 | 22 |
| qctFad 2.2-A1 | CAAGATGGATGCGATGGTAAGG | 67.3 | 22 |
| qctFad2.2-S1 | GCCTCCAAAGATTCATTCAGGTC | 66.7 | 23 |
| qctKas II-A1 | GGTATTGGTATTGGATGGGCG | 66.8 | 21 |
| qctKasII- S1 | CCTAGATAATTGCAGTTCAGATG | 59.3 | 23 |
| qctFad 2.1-S1 | GTGTATGTCTGCCTCCGAGA | 62.9 | 20 |
| qctFad 2.1-A1 | GCAAGGTAGTAGAGGACGAAG | 59.5 | 21 |


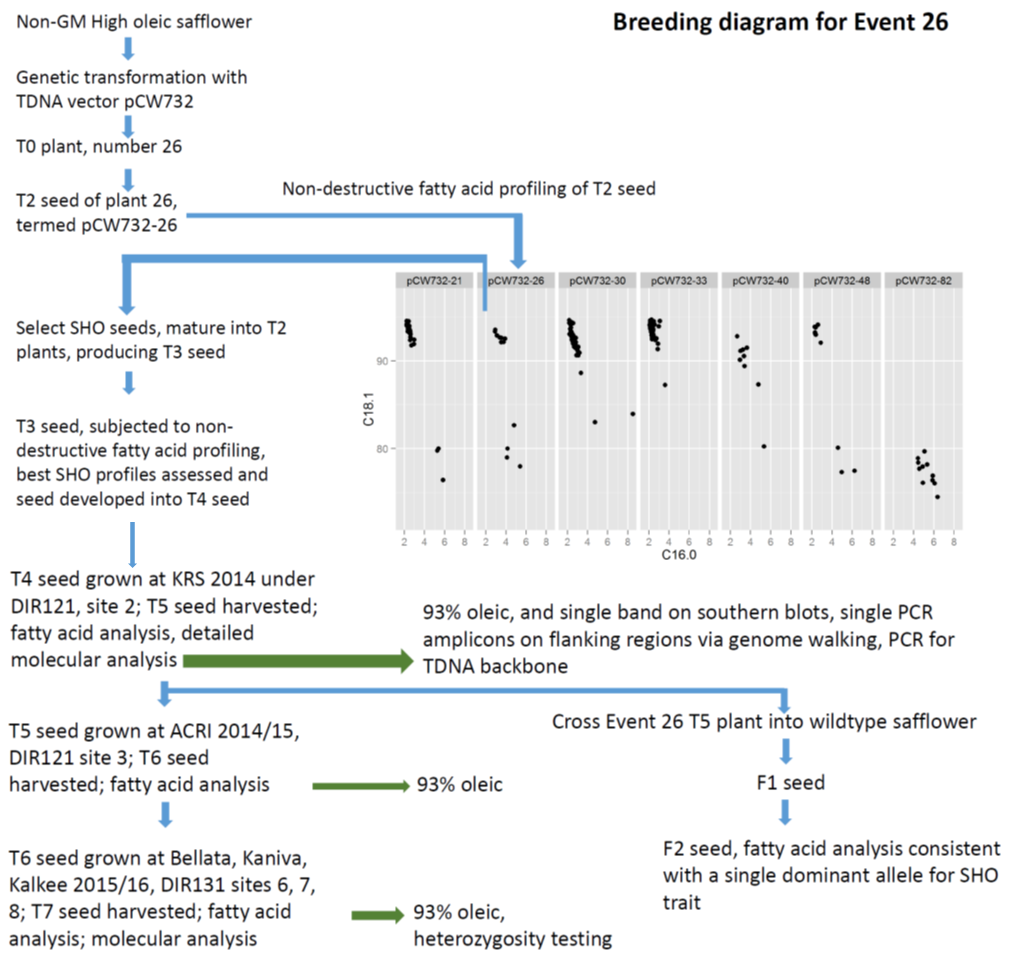


Supporting Figure 1: A schematic outlining the biogenesis of SHO Events outlined in this report, using Event 26 as an example. The diagram outlines the key agronomic milestones and molecular assays used and their outcome, as outlined elsewhere in the study.


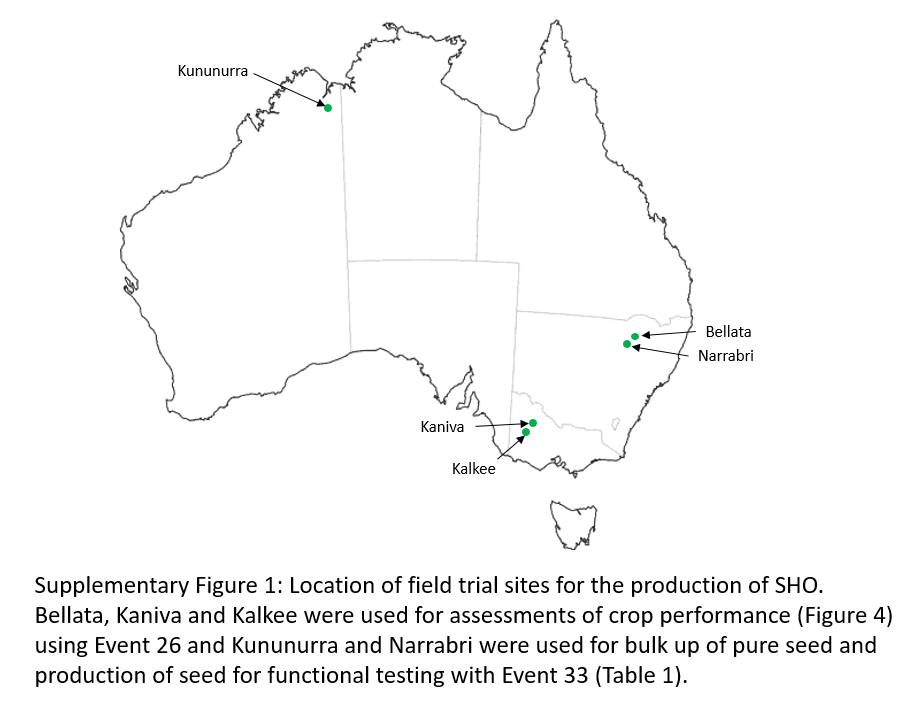


Supporting Information 2: Location of field trials for the production of superhigh oleic safflower. Kununurra (KRS, 2014) and Narrabri (ACRI, 2014-15) were used for bulk up of pure seed of Event 33 sufficient for functional testing while sites Bellata, Kaniva and Kalkee (2015-2016) were used for assessments of crop performance of Event 26.
